# Computed Tomography to Estimate the Representative Elementary Area for Soil Porosity Measurements

**DOI:** 10.1100/2012/526380

**Published:** 2012-05-01

**Authors:** Jaqueline Aparecida Ribaski Borges, Luiz Fernando Pires, André Belmont Pereira

**Affiliations:** ^1^Laboratory of Soil Physics and Environmental Sciences, Department of Physics, State University of Ponta Grossa (UEPG), Avenue Carlos Cavalcanti 4748, 84030-900 Ponta Grossa, PR, Brazil; ^2^Department of Soil Science, State University of Ponta Grossa (UEPG), Avenue Carlos Cavalcanti 4748, 84030-900 Ponta Grossa, PR, Brazil

## Abstract

Computed tomography (CT) is a technique that provides images of different solid and porous materials. CT could be an ideal tool to study representative sizes of soil samples because of the noninvasive characteristic of this technique. The scrutiny of such representative elementary sizes (RESs) has been the target of attention of many researchers related to soil physics field owing to the strong relationship between physical properties and size of the soil sample. In the current work, data from gamma-ray CT were used to assess RES in measurements of soil porosity (*ϕ*). For statistical analysis, a study on the full width at a half maximum (FWHM) of the adjustment of distribution of *ϕ* at different areas (1.2 to 1162.8 mm^2^) selected inside of tomographic images was proposed herein. The results obtained point out that samples with a section area corresponding to at least 882.1 mm^2^ were the ones that provided representative values of *ϕ* for the studied Brazilian tropical soil.

## 1. Introduction

Computed tomography (CT) is proven an efficient technique that can be largely used in studies related to soil structure [1–3]. It has been seen as an important tool to be adopted by new generation's tomographs designed exclusively for research carried out with porous materials [4, 5]. The success of the aforementioned technique is ascribed to a method that is noninvasive to determine physical properties in a cross-section of a material. Another advantage of such technique is that CT also provides 2D and 3D images with micro- and millimetric resolutions and allows qualitative and quantitative analyses [6].

 Among several practical applications [7–9], CT is also an excellent technique employed to assess representative sizes of soil samples, as well as to scrutinize soil physical properties. This is because it is possible to select volumes, areas, or lengths of different sizes in the inside of tomographic images, depending on the generation of the equipment [10, 11].

 The concept of representative elementary size (RES) was first introduced to the continuum mechanics by Jacob Bear in 1972 as a tool to be employed to describe flow in porous media. The approach deals with the definition of a minimal size or physical point of a sample necessary for representing its characteristics of interest. In other words, it refers to as the size at which a measured parameter turns out to be independent of the size of the sample [12].

**Figure 1:**
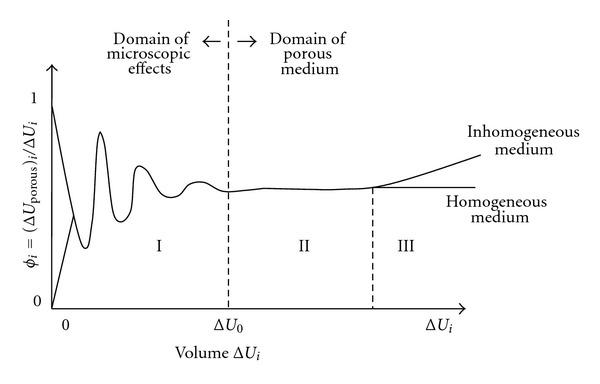
Representative elementary volume (REV, Δ*U*_0_) for the porosity (*ϕ*). Δ*U*_*i*_ represents any volume in the porous media. REA is defined from the concept of REV and shows similar behavior. Source: adapted from Bear [12].

 The analysis applied to RES is commonly made by selecting consecutive sizes around a central point in the image of the sample. It is reported in the literature that adjacent selections within the same image and centered in different points can also be utilized [11, 13]. The representative size is then defined as that one corresponding to the domain transition of the microscopic effects (region I) to the domain of a porous media (region II) (Figure 1).

The main concern with the use of samples with representative sizes is due to the relationship between soil physical properties and size of soil samples [14, 15]. However, such sizes are normally investigated for properties of a particular interest in homogeneous media, such as spherical glass beads and sands [10]. Moreover, representative elementary volume (REV) in particular became a parameter that demonstrates the quality of the measurements made via third-generation CTs [13].

The available research in the literature for nonhomogeneous media with porosity and/or particles size varying in space is still scarce. In these cases, a difficulty for determining the elementary size is observed under experimental conditions whenever slight fluctuations occur for the analyzed physical property. In order to overcome such a problem, statistical tools have been applied to delimitate the maximum allowable variation in each case, setting up therefore, a reliability level for RES [15].

Porosity (*ϕ*) is an important index that reveals the structural quality of a soil and represents the volume of a soil not occupied by solid particles, including all porous spaces occupied by water and air. Porosity is fundamentally linked to the root growth and movement of air, water, and solutes in the soil. For instance, a well-structured soil generally possesses intraaggregate (textural) and interaggregate (structural) pores, being the macroporosity an index that expresses the structural quality of the soil. Soils with a good quantity of macropores (10%) will favor the gas exchanges and the development of the root system for crop production [16].

Faced with the scarcity of published manuscripts that deals with representative sizes for nonhomogeneous samples in conjunction with the agricultural relevance of representative measurements of soil porosity, the current contribution aims to make use of computed tomography to determine the representative elementary area for measurements of *ϕ*.

## 2. Materials and Methods

### 2.1. Soil Sampling

Eighteen soil samples were collected from an experimental field belonging to the University of São Paulo-(ESALQ/USP)-located at Piracicaba, SP, Brazil (22°42′S and 47°38′W, 580 m above sea level). Samples were collected in triplicate, being selected 6 collection points along a transection of 200 m long. The volumes of the soil clod sample varied from 50 to 100 cm^3^. The sampling was made at the surface layer (0–15 cm) inside of a small trench. Shortly before the opening of a trench the crop above the soil surface was removed.

The clay soil (43% clay, 24% sand, and 33% silt) was classified as an Eutric Nitosol [17]. It presents a soil particle density (*ρ*_p_) of 2.65 g cm^−3^. As to its chemical characteristics, the soil possesses 20.2 g dm^−3^ of organic matter; pH 5.3 (in CaCl_2_), and 29.0, 20.0, and 4.3 mol m^−3^ of Ca, Mg, and K [18].

### 2.2. Gamma-Ray Computed Tomography

CT utilized is a first-generation scanner with the source and detector fixed and with rotation and translation movement of the sample. The scanner was built by engineers from EMBRAPA/CNPDIA (São Carlos, Brazil) and presents the following modules: (1) ^241^Am (59.54 keV, 3.7 GBq) gamma ray source mounted in a Pb shield castle; (2) Nal(Tl) scintillation crystal detector (7.62 × 7.62 cm) coupled to a photomultiplier tube; (3) electronic modules (preamplifier, high-voltage supplier, single channel analyzer, counter and timer); (4) step motor (rotational and translational movements); (5) Pb collimators; (6) software for CT data acquision [19]. The counter, with an RS-232 interface, makes communication with an IBM PC that controls the step motor.

Lead collimators with 1 and 4.5 mm were placed in front of the source and detector to collimate the beam. The matrixes of tomographic units (TUs) data obtained were of 80 × 80 for all tomographs. The resolution obtained for the clod samples was of 1.1 × 1.1 mm^2^. A 2D section image for each clod was obtained at the center of the sample.

TU is proportional to the linear attenuation coefficient, *μ* (cm^−1^), and the reference media for the TU is the air, showing the lowest attenuation indices. For the soil system, TU corresponds to the contribution of the mineral particles, organic matter, water, and air, generating different values of *μ* for each crossed sample path by the radiation beam [20].

The relationship between TU and different physical properties of the soil, such as soil bulk density (*ρ*_s_) and its volumetric water content (*θ*) is given by (1) [3, 21, 22]:

(1) $$\text{TU}=\alpha \left(\mu_{\text{ms}}\rho_{\text{s}}+\mu_{\text{mw}}\rho_{\text{w}}\theta \right),$$

where *ρ*_w_ (g cm^−3^) is the water density, *α* is the angular coefficient of the calibration straight line of the tomographic system, *μ*_ms_  and *μ*_mw_ (cm^2^ g^−1^) are the mass attenuation coefficients of soil and water, respectively.

It is possible to calculate *ϕ* for each TU data of the dry soil sample scanned by using a combination of the equation used to determine *ϕ* by conventional methods [23] and (1) as follows:

(2) $$\phi =\left(1-\frac{\rho_{\text{s}}}{\rho_{\text{p}}}\right)=\left(1-\frac{\text{TU}}{\alpha \mu_{\text{ms}}\rho_{\text{p}}}\right).$$

For the calibration of the scanner, samples of the following homogeneous materials were used: acrylic, ethanol, water, nylon, and glycerin. 2D section images were obtained at the center of samples used for calibration. A thorough description of the calibration process of the first-generation scanner can be found in Crestana et al. and Pires et al. [24, 25].

### 2.3. Attenuation Coefficient

In order to evaluate the soil linear attenuation coefficient (*μ*_s_), samples were air dried and sieved with a 2.0 mm mesh. After sieving, the soil was transferred to an acrylic box with the dimensions of 4.9 × 5.1 × 5.5 cm. The intensities of monoenergetic photons were obtained at three different positions of the acrylic box filled with soil. Five replicates were taken for each position. The same procedure was performed to assess the linear attenuation coefficient of water (*μ*_w_).

The mass attenuation coefficients of soil and water were calculated dividing the experimental *μ* by the *ρ* of the samples (*μ*_m_ = *μ*/*ρ*). For the case of water, the density was considered as *ρ*_w_ = 1 g cm^−3^.

### 2.4. Representative Elementary Area

Matrixes of TU data obtained via CT (80 × 80) were firstly converted into density and porosity matrixes by means of (1) and (2). The images were reconstructed using the software Microvis [26]. Darker regions in the images correspond to the lowest values of density and, consequently, to the lowest values of TU. In the current work, the darker regions represent larger values of density.

For the REA evaluation, the largest possible rectangular area at the center of the sample was delimitated with no interference of the edges in a tomographic image. The edges were avoided since the interface sample-air might generate artifacts that can affect the analysis of soil physical properties by CT [19, 27].

**Figure 2:**
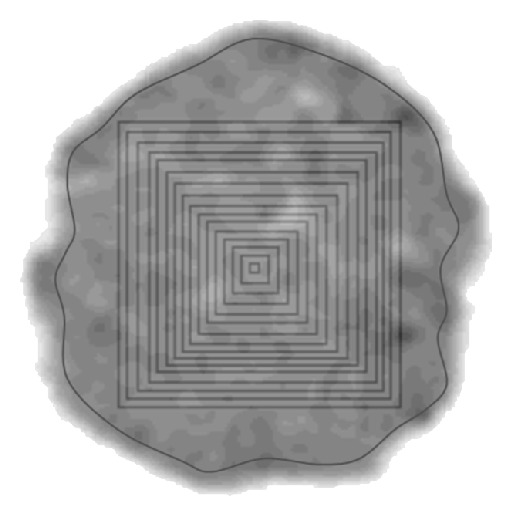
Schematic drawn of the area construction on the tomographic images. The area next to edge corresponds to the free area (FA). Darker regions represent higher soil bulk density values.

The reference points at each vertex of a rectangular area were selected in the tomographic image by means of Microvis software and identified and demarked in the matrix of TU afterwards. Then, consecutive concentric quadrangular areas (Figure 2) were selected without extrapolating the maximum area previously chosen. The initial area was obtained from a 1 : 1 square matrix (1.1 mm × 1.1 mm, Table 1). The number of delimited areas for each sample image varied in compliance with its size and differences in its shape. Plus an area with an irregular shape containing almost the entire tomographic image was also selected to refer to the free area (FA).

The linear steps of the tomographic system were of 1.0 to 1.1 mm. The discrepancies in the linear steps are due to different dimensions of the samples of the analyzed soil.

 The *ϕ* was determined for each one of the quadrangular areas, as well as for the FA. Soil porosity obtained via CT corresponds to the mean value of such a physical property once CT allows for its analysis point by point (from “pixel” to “pixel”).

**Figure 3:**
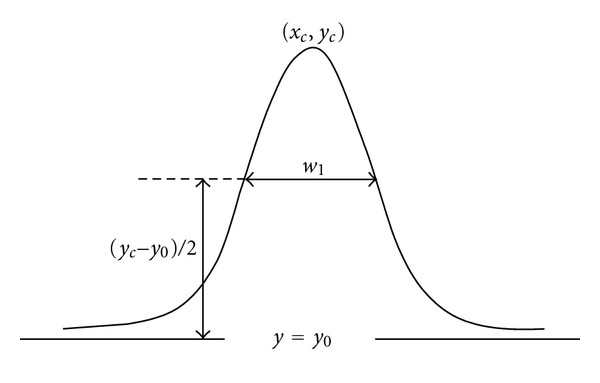
Schematic representation of the parameters used to calculate the full width at a half maximum (FWHM) for a normal distribution. Adapted from Origin User Guide [28].

 The identification of REA was established taking into account the criterion used by Vandenbygaart and Protz [15]. Below are listed the analyses made and the criteria adopted to define REA as a function of *ϕ*:

- determination of *ϕ* values frequency within each area, for the FA, the computed frequency corresponds to the interval of porosity present in the selected FA for each sample;
- elaboration of graphs that show the frequency of *ϕ* (%), such a procedure was performed for each one of the areas;
- determination of the full width at a half maximum (FWHM) of each distribution by means of Gaussian adjustments, which were obtained from the fifth area of each sample (matrix 9 × 9), in the present study, the parameter FWHM was used to describe the distribution of *ϕ* of the samples in each area, the adjustment equation is
  (3) $$y_{c}=y_{0}+\frac{\text{A}}{w\sqrt{\pi /2}}e^{-2{\left(x-x_{c}\right)}^{2}/w^{2}},$$
  where *y*_*c*_ is the maximum height of the adjustment curve; *y*_0_ is the basis of the curve; A is the area below the curve; *w* is the parameter given by
  (4) $$w=\frac{w_{1}}{\sqrt{\ln\left(4\right)}},$$
  where *w*_1_ is the FWHM of the curve; *x* is any position in the abscissas axis and *x*_*c*_ the central position of the adjustment curve in the same axis (Figure 3);
- determination of relative deviations between the value of FWHM corresponding to the distribution of *ϕ* for the last rectangular area and each one of its previous areas;
- REA for distribution of *ϕ* is reached whenever three consecutive areas did not present deviations higher than 10%;
- graphs for the values of FWHM for each area of the samples (including FA) were made, for the samples that reached REA, corresponding areas were demarked.

## 3. Results and Discussion

**Figure 4:**
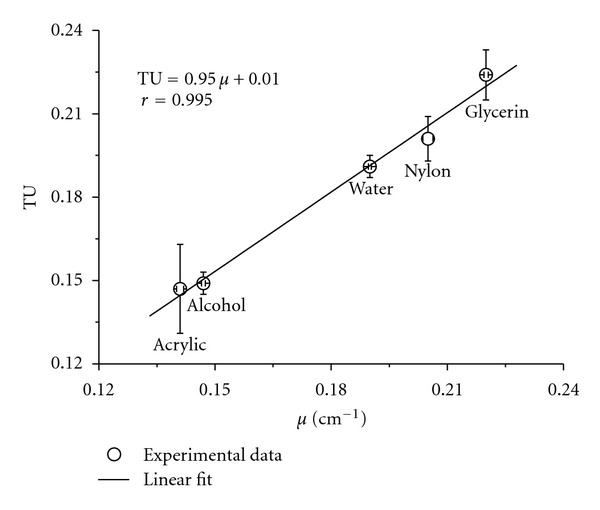
Experimental relationship between the tomographic units (TU) of the images obtained by the tomograph and linear attenuation coefficients (*μ*) for homogeneous substances used during the calibration of the tomograph. The vertical error bars represent the standard deviation of the TU values in the matrix of selected data. The horizontal error bars depict the mean standard deviation (*n* = 3).

The coefficient of correlation (*r*) obtained during the calibration of the CT system was of 0.995 (Figure 4). Such a good correlation between the experimental data is of a great importance in studies conducted to acquire representative measurements of soil physical properties through CT [25].

The *μ*_ms_ and *μ*_mw_ values were 0.3339 ± 0.0029 and 0.2001 ± 0.0004 cm^2^ g^−1^, respectively. Such values are coherent when compared to the experimental and theoretical outcomes obtained by other researchers for water and soil with the same texture of that one studied herein [29, 30].

**Figure 5:**
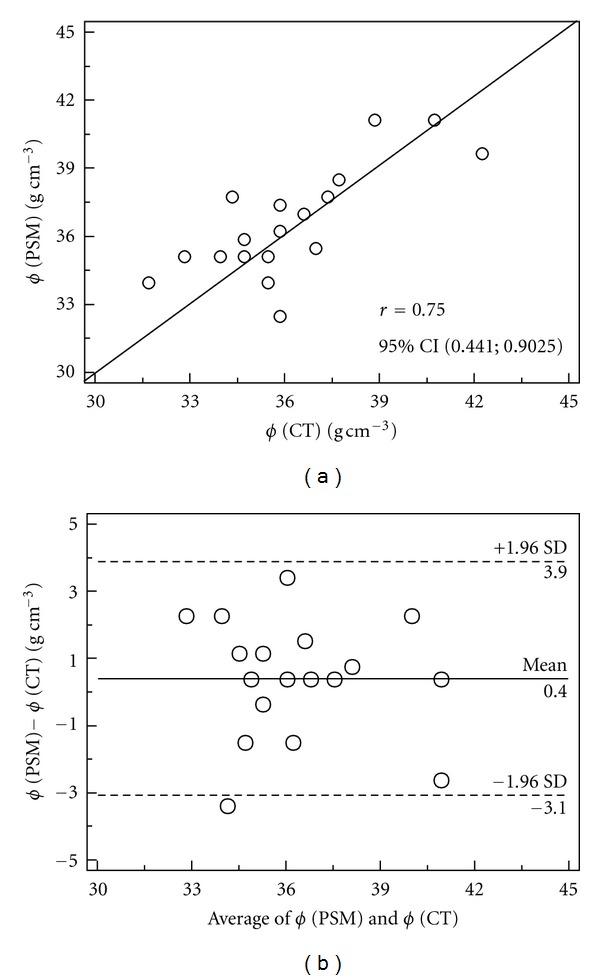
(a) Soil porosity (*ϕ*) measured by the computed tomography (CT) and paraffin-sealed methods (PSMs), with line of equality; (b) Bland-Altman plot.

The *ϕ* values calculated by the CT technique were compared to the values obtained by the paraffin-sealed method (PSM) for the same soil (Figure 5). The mean values of *ϕ* obtained by means of PSM and CT were of 37 and 36%, respectively. A part from that, a correlation study between methods was performed (*r* = 0.75) and a relatively strong positive correlation was observed between the variables in study (Figure 5(a)). The Bland-Altman analysis [31] was also performed to reveal the relationship between the differences and the magnitude of measurements (Figure 5(b)). A good agreement was found since the mean difference is close to zero and not statistically significant (test statistic *t*, 0.05). From the plot, it is also possible to observe that 1/18 (5.5%) of the points are beyond the limits of agreement (±2sd lines).

**Figure 6:**
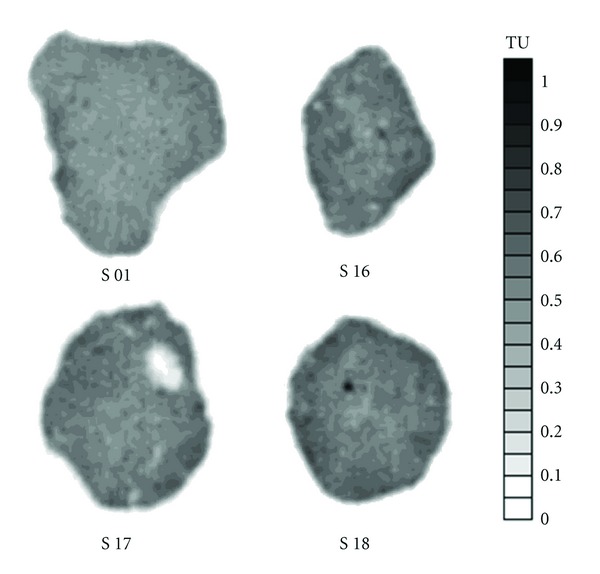
Tomographic images referent to the samples 01, 16, 17, and 18. The images are in a grey scale of tomographic units (TU), where the darker regions indicate the highest value of TU.

 In Figure 6, we can visualize tomographic images of some studied samples. Besides being capable of conducting quantitative studie on soil physical properties via CT images, we can also have a qualitative idea of its spatial variability. For instance, S 01 (Figure 6) does not show great discrepancies on its structure, being more homogeneous (see grey scale) in relation to the other samples.

By elaborating graphs for frequency distribution of *ϕ* in each selected area of images, we observed that such images presented a Gaussian distribution of *ϕ* from the fifth selected area. Such a fact was explained in compliance with the central theory of the limit, which assumes that each randomized variable with no particular distribution approaches the normal in so far as the sample size increases [32].

**Figure 7:**
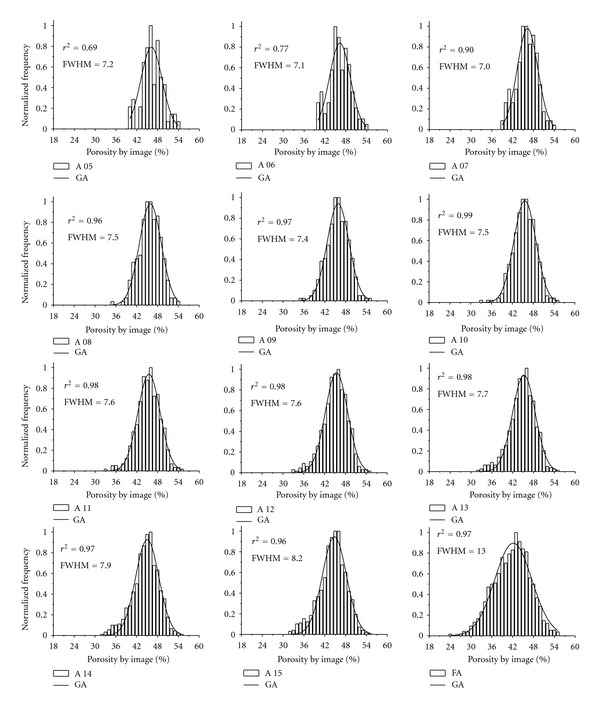
Normalized frequency of the porosity by image (%) from the 5th selected area in the sample 01 (S 01). A 05, A 06,…, and A 15 correspond to the sequence of quadrangular areas selected in the image and free area (FA). GA represents the Gaussian adjustment, *r* ^2^ the coefficient of determination, and FWHM the full width at a half maximum of the curve.

**Figure 8:**
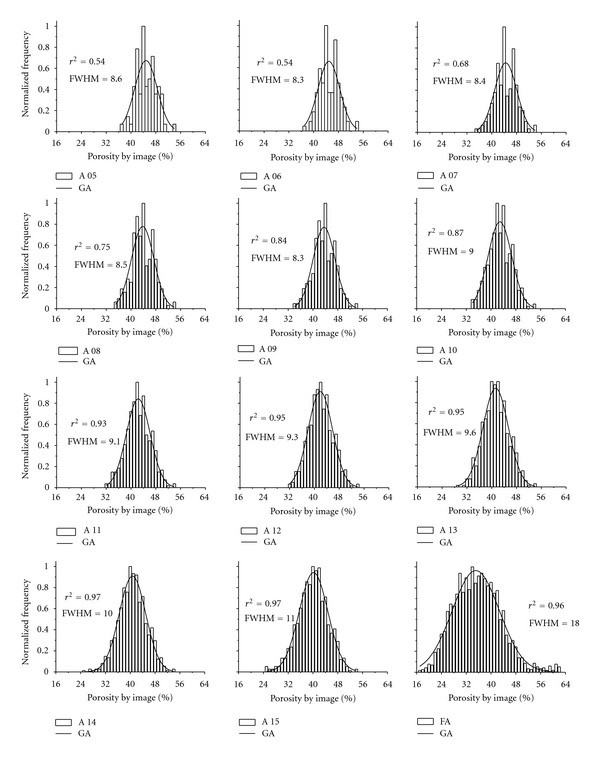
Normalized frequency of the porosity by image (%) from the 5th selected area in the sample 10 (S 10). A 05, A 06,…, and A 15 correspond to the sequence of quadrangular areas selected in the image and free area (FA). GA represents the Gaussian adjustment, *r* ^2^ the coefficient of determination, and FWHM the full width at a half maximum of the curve.

Figures 7 and 8 show the distributions obtained experimentally for S 01 and S 10. The sample S 01 (Figure 7) reveals that the superior limit of the distribution of *ϕ* remains roughly the same, whereas the inferior limit turns from 40% (5th area) to 24% (FA) of *ϕ*. On the other hand, for S 10 (Figure 8), such an inferior limit varies from 37% to 17% of *ϕ*. The superior limit of the distribution of *ϕ* for S 10 also shows a great variation, from 54% to 62%. However, such variations at the extremities of the curves have a despicable influence on FWHM of the distribution, since this variable is directly influenced by the distribution of the central values. Although FWHM comes to being normally used for resolution measurements in spectral analysis, we opted to use it in the current study aiming at generating a new parameter to predict REA for *ϕ*.

 The coefficient of determination (*r* ^2^) obtained for each one of the distributions is quite high. Its value is equal or greater than 0.90 in 16 out of 24 distributions presented—a fact that indicates a good agreement between the variables studied.

 The central position of the distribution at the abscissas axis (*x*_*c*_) demonstrated a slight variation within the same sample for consecutive areas. However, the variation is more pronounced when we compare FA for previous areas. This happens because the FA shows an interval of a large distance of the quadrangular areas, whilst they were selected consecutively. Moreover, FA includes practically the entire sample, which causes the heterogeneity of it to be greater than that one of small areas.

**Figure 9:**
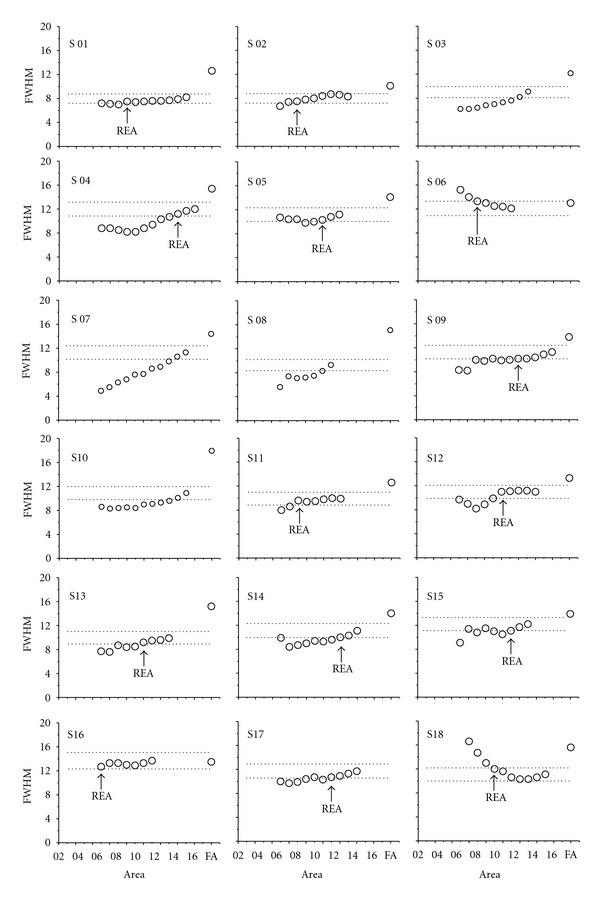
Graphs to depict values of FWHM from the 5th area selected in 18 samples (S 01, S 02,…, and S 18) and its respective representative elementary areas (REA) for a deviation of 10% when the REA was reached.

Figure 9 shows the graphs for FWHM from the 5th area selected in 18 samples and its respective REA for a deviation of 10%, when REA was actually reached. The curves obtained for four samples in Figure 6 might be better explained by analyzing the variability of *ϕ* for such samples.

Taking the criterion of variation adopted (item 2.4) into consideration, only 14 out of 18 samples reached the REA for *ϕ*. Such a fact might be explained by the spatial variability presented by this physical property [33]. For instance, in order to compare it to *ρ*_s_, the intervals of variation coefficients (CV) of *ϕ* and *ρ*_s_ are 7–11% and 3–26%, respectively. The interval of CV for *ρ*_s_ is higher; however, its inferior limit is smaller than the inferior limit of *ϕ*.

 The different values of REA found in this study might be ascribed to a nonhomogeneity of the samples. For instance, Pires et al. [34] analyzed the different values of TU obtained within the same sample, divided into 15 adjacent areas. The authors observed a significant variation among TU, a point that brought about variations on soil structure as a result of natural or artificial processes. The largest difference obtained between the areas of the same sample was of 111 TU, whereas the smallest one corresponded to 40 TU.

 In the current work, we observed small stones and/or big voids (biopores) in some of the samples studied, while others are in its totality denser in relation to the others (e.g., S 14 and S 18). Such characteristics of each sample could be visualized and quantified in the tomographic images in such a nondestructive way, a procedure that might not be adopted by making use of traditional methods to measure *ϕ*.

Among the eighteen cases studied herein, only the S 16 did not show a crescent FWHM in relation to FA. However, the difference between FWHM of the distribution of *ϕ* of the last analyzed area and FA of this sample is just 0.2. This might be attributed to the hypothesis that such a sample probably presents a high value of *ϕ* in its central region (Figure 6). Thus, the variation of *ϕ* is already included in smaller areas, causing just an increase in frequency of the central values as the counting is made for FA. With regard to S 17, it was not possible to insert the point referent to FA. This can be explained due to the distribution of frequency, which presented a bimodal behavior for this area and, therefore, impaired the measurement of FWHM for FA by means of the criteria adopted for the other areas.

**Figure 10:**
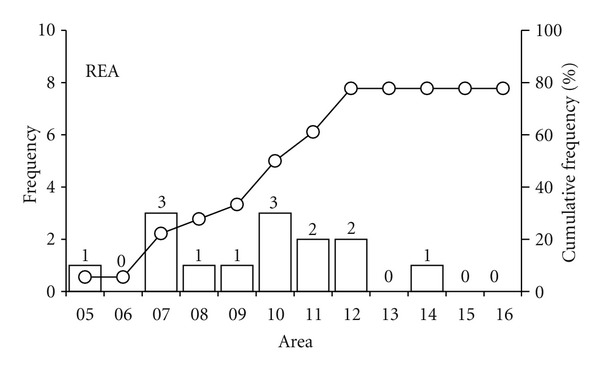
Graphs of full width at a half maximum (FWHM) values from the 5th selected area in 18 samples and its respective representative elementary areas (REA) for a 10% deviation, when REA was reached.

Following up the 10% variation criterion, as adopted by Vandenbygaart and Protz [15], the 14 samples that reached the REA were the ones that did it so up to the 14th area (Figure 10). In this case, samples with a section area of at least 882.1 mm^2^ give representative values of *ϕ*.

It is important to notice that the representative minimum size of an area may vary as a function of the physical property investigated and also according to the material utilized. For instance, by Al-Raoush and Papadopoulos [14] such a point was discussed and they reached the conclusion that a study carried out to look at the porosity of an analyzed media cannot be used as a basis for representative measurements of other parameters of interest. This is because other parameters require a size large enough to provide representative measurements. Thus, the usage of the same representative size for measurement of different properties is going to be dependent on the fact whether or not it includes REA in all parameters analyzed.

It is important to mention that although this work presents results for only one type of soil (clay soil); measurements for different soil types can be easily made. However, it is necessary to obtain the TU data matrix, after CT scanning, in which the different areas to determine RES will be selected. Another condition for the reproducibility of this study is the sample size. Samples larger than those used in this work can present artifacts due to the excessive attenuation of the ^241^Am radiation beam. Low spatial resolution will also be obtained for large samples affecting the quality of RES analysis [13].

## 4. Conclusions

CT employed to assess REA made possible the distribution of soil porosity (*ϕ*) to be analyzed in both qualitative and quantitative terms. The study of FWHM of the adjustment curves of the distribution of *ϕ* for crescent selected areas in tomographic images showed to be satisfactory to determine REA.

FWHM expresses the distribution of *ϕ* prevailing in the investigated area, since the extreme values of such property (samples with small stones) cause insignificant implications in its value. However, when sample presents a region with stones and macropores, the distribution of *ϕ* might turn out to be bimodal and difficulties on the analysis of FWHM can be found.

Faced with the evaluation employed for REA, the results indicate that samples with sizes of at least 882.1 mm^2^ provided the representative values of *ϕ* for the investigated soil.

## Figures and Tables

**Table 1:** Areas (mm^2^) adopted for the REA definition.

Area	Size (mm^2^)	Area	Size (mm^2^)	Area	Size (mm^2^)	Area	Size (mm^2^)
01	1.2	05	98.0	09	349.7	13	756.3
02	10.9	06	146.4	10	436.8	14	882.1
03	30.3	07	204.5	11	533.6	15	1017.6
04	59.3	08	272.3	12	640.1	16	1162.8
